# Astilbin: a novel therapeutic strategy for bone destruction in rheumatoid arthritis

**DOI:** 10.3389/fimmu.2026.1932692

**Published:** 2026-09-09

**Authors:** Fuyuan Zhang, Yuchen Yang, Xieli Ma, Jun Li, Congmin Xia, Jian Wang, Quan Jiang

**Affiliations:** 1 Guang’anmen Hospital, China Academy of Chinese Medical Sciences, Beijing, China; 2 Postdoctoral Station of China Academy of Chinese Medical Sciences, Beijing, China

**Keywords:** astilbin, bone destruction, inflammation, MAPK3, osteoclast, rheumatoid arthritis

## Abstract

**Background:**

Bone destruction constitutes the primary cause of disability in rheumatoid arthritis (RA). Herbal medicines feature unique multi-target advantages, rendering them promising therapeutic candidates for attenuating RA-associated bone damage.

**Objective:**

This study aimed to investigate the therapeutic efficacy and underlying mechanisms of astilbin, a core bioactive flavonoid extracted from Tufuling (Smilax glabra), against bone destruction in RA.

**Methods:**

A CIA mouse model was established via tail vein injection of type II collagen. Micro-CT, HE staining and TRAP staining were applied to evaluate the inhibitory and therapeutic effects of astilbin on RA bone destruction. Network pharmacology and bioinformatics analyses were performed to screen the potential targets and signaling pathways of astilbin for RA treatment. Mendelian randomization analysis was further conducted to genetically validate the correlation between candidate genes and RA. Molecular docking was utilized to calculate binding energies between astilbin and core pathogenic targets of RA. Immunofluorescence, elisa, WB and qPCR were adopted to verify the key targets and anti-inflammatory activity of astilbin in ameliorating RA bone erosion.

**Results:**

*In vivo* animal experiments demonstrated that astilbin alleviated RA bone destruction, suppressed synovial hyperplasia, inhibited osteoclastogenesis, and downregulated the expression of pro-inflammatory cytokines. Network pharmacology identified MAPK3, MAPK1, PTPN11, HSP90AA1, STAT1 and SRC as the core therapeutic targets of astilbin against RA. KEGG enrichment analysis revealed that the anti-RA effects of astilbin were tightly associated with the MAPK signaling pathways and so on. Mendelian randomization analysis further verified a strong genetic correlation between the apoptotic protein MAPK3 and RA. Molecular docking results exhibited favorable binding affinity between astilbin and key pathogenic targets of RA.

**Conclusion:**

Astilbin may exert multiple pharmacological effects including anti-inflammation, inhibition of osteoclast activation, improvement of bone microarchitecture and alleviation of bone destruction in RA via regulating the MAPK signaling pathway, and it exhibits favorable safety *in vivo*.

## Graphical Abstract

**Figure:**
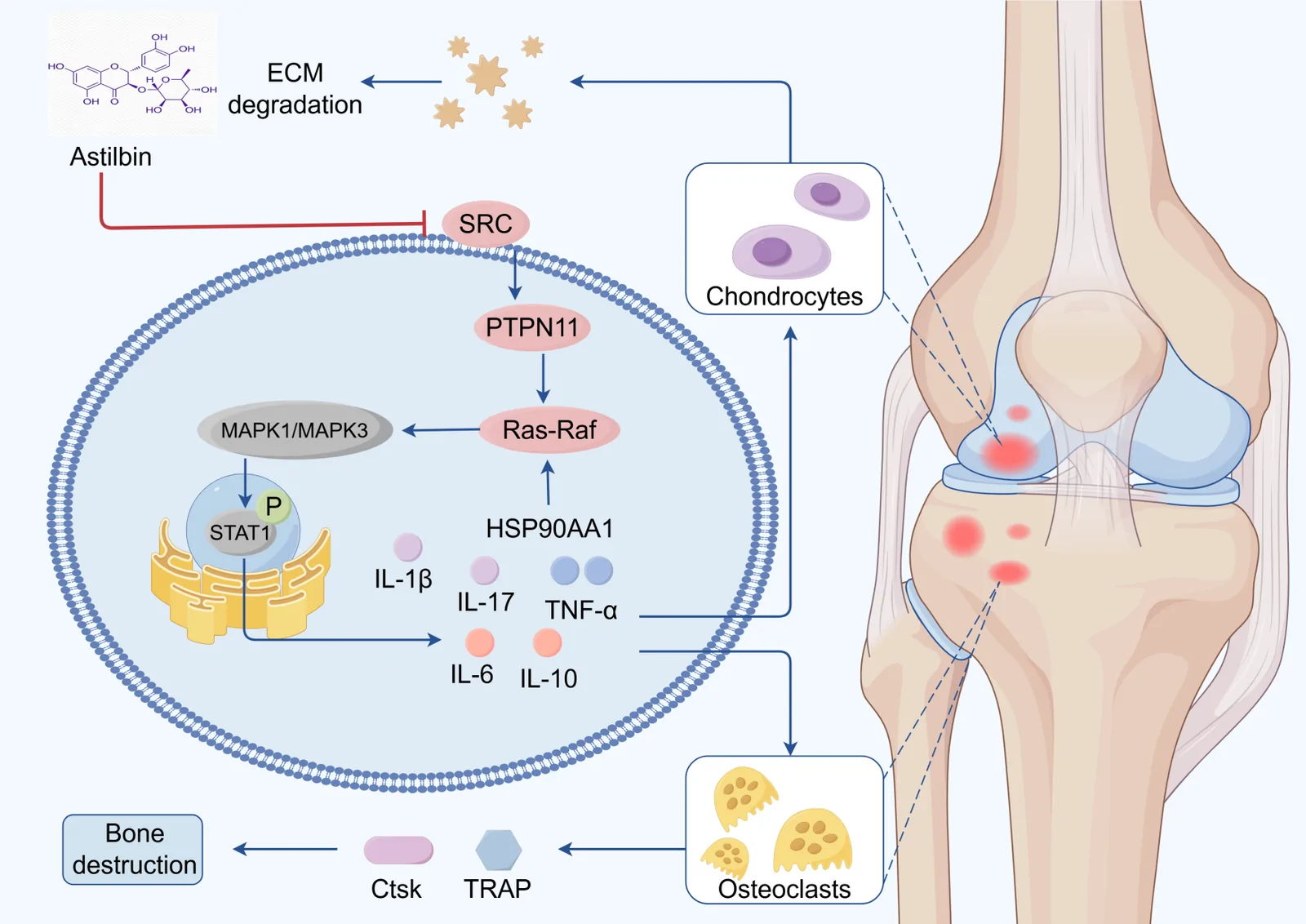
Astilbin suppresses osteoclastogenesis and pro-inflammatory cytokine secretion by multi-target regulation of the MAPK signaling cascade, improves the disordered synovial microenvironment, and ultimately delays pathological bone destruction in RA.

## Highlights

- Astilbin exerts prominent anti-inflammatory and bone-protective effects to alleviate synovial hyperplasia, inflammatory infiltration and progressive bone destruction in RA.
- Via multi-target regulation, especially by targeting MAPK3, astilbin blocks the temporal progression of RA from early inflammation to irreversible late stage bone erosion, with favorable in vivo safety.
- The therapeutic effects of astilbin against RA are closely associated with the MAPK signaling pathway.

## 1 Introduction

Rheumatoid Arthritis (RA) is a disabling inflammatory arthropathy characterized by systemic chronic autoimmune disturbance, clinically known as “incurable cancer” (1). Its global incidence and prevalence have risen year by year, imposing a severe disease burden (2). The pathological progression of RA consists of two core links: chronic inflammatory infiltration of the synovium and abnormal pannus proliferation. Irreversible bone and cartilage destruction represents the terminal pathological change that determines joint deformity, loss of limb function and impaired long-term quality of life in patients (3, 4). It also remains a bottleneck difficult to resolve in current clinical management.

Current therapeutic regimens for RA mainly include nonsteroidal anti-inflammatory drugs (NSAIDs), conventional synthetic disease-modifying antirheumatic drugs (csDMARDs) and biological agents (5). Although these medicines can partially suppress systemic inflammation and relieve joint swelling and pain, they have prominent limitations. On one hand, most drugs fail to specifically block excessive osteoclast activation or reverse established bone erosion, making it hard to fundamentally retard the progression of bone destruction (6). On the other hand, long-term administration tends to trigger toxic side effects such as gastrointestinal injury, hepatic and renal dysfunction, and myelosuppression (7). Furthermore, patients are prone to develop drug tolerance after continuous medication, accompanied by inflammatory rebound and progressive bone destruction, which greatly restricts long-term clinical benefits (8). Accordingly, developing novel bioactive molecules with dual anti-inflammatory and bone-protective effects, low toxicity and low drug resistance has become an urgent research hotspot in the field of rheumatology and immunology.

Chinese herbal medicines, with unique advantages such as multi-target characteristics and favorable safety, have emerged as potential therapeutic strategies for RA (9). Accumulating evidence demonstrates that active ingredients derived from certain Chinese herbal medicines can modulate mitochondrial function and regulate oxidative stress, thereby exerting anti-synovitis and anti-apoptotic effects (10, 11). Astilbin is the predominant bioactive monomer isolated from Tufuling (Smilax glabra Roxb). Studies have verified that astilbin suppresses the expression of pro-inflammatory cytokines and ameliorates RA in combination with methotrexate (MTX) (12, 13). Nevertheless, current available studies merely focus on its simple anti-inflammatory phenotype. Systematic elucidation of mechanisms underlying RA-characteristic bone destruction remains insufficient, and the pivotal target genes and molecular pathways by which astilbin regulates bone homeostasis have not yet been clarified. Meanwhile, most existing investigations are restricted to a single animal model. A complete multi-dimensional evidence chain, covering genetic causality, network pharmacology prediction, molecular interaction verification and *in vivo* pharmacodynamic evaluation in animals, is still lacking to support the mechanism of astilbin against bone destruction in RA.

Based on the holistic concept of systems biology, network pharmacology overcomes the limitations of conventional single-target screening approaches (14–16). It enables large-scale identification of all potential target genes of small-molecule compounds and systematic mapping of upstream and downstream signaling regulatory networks. As a mature research tool for deciphering the mechanisms of Chinese herbal medicines, crude extracts and phytochemical monomers, network pharmacology provides a standardized analytical framework to explore the action mechanisms of natural bioactive constituents (17, 18).

Therefore, this study first adopted multiple databases for network pharmacology to screen potential shared targets of astilbin against RA, and constructed a protein-protein interaction (PPI) network to identify core targets. Mendelian randomization (MR) analysis was applied to verify a stable causal association between MAPK3 expression and RA pathogenesis at the genetic level. Molecular docking was performed to validate the binding capacity of astilbin to key targets of RA. Finally, a collagen-induced arthritis (CIA) mouse model was established, and *in vivo* pharmacodynamic evaluation was carried out via micro-CT analysis of bone microarchitecture, histopathology, tartrate-resistant acid phosphatase (TRAP) staining, western blot (WB), quantitative real-time polymerase chain reaction (qPCR), immunofluorescence, and elisa detection of serum inflammatory cytokines. This study aims to provide a novel therapeutic strategy for RA.

## 2 Materials and methods

### 2.1 Identification of potential targets for the treatment of RA with astilbin

The 2D structure (mol2 file), CAS number, and smile number of astilbin were downloaded from PubChem (https://pubchem.ncbi.nlm.nih.gov/). Drug targets were obtained by entering “astilbin” into the Comparative Toxicogenomics Database (https://ctdbase.org/), mol2 file into Pharmmapper Database (http://lilab-ecust.cn/pharmmapper/), smile number into Swisstarget Database (http://swisstargetprediction.ch/), and CAS number into Traditional Chinese Medicine Systems Pharmacology Database (https://old.tcmsp-e.com/tcmsp.php). Species were selected “human” and the probability values greater than 0. The drug targets obtained from the above databases were intersected and duplicates were removed.

In the GeneCards Database (https://www.genecards.org/), “RA” was used as the main search term, and the relevance score was limited to greater than 1 to obtain the disease targets of RA. Further, we searched for disease targets of RA in Therapeutic Target Database (https://db.idrblab.net/ttd/), PharmGKB Database (https://www.pharmgkb.org/), and Online Mendelian Inheritance in Man (https://www.omim.org/). All the disease targets of RA obtained above were removed duplicates and intersected. We intersected the drug targets and disease targets above to obtain potential targets for the treatment of RA by astilbin. And venn figure was drew by R software.

### 2.2 PPI network construction and enrichment analysis

In order to construct Protein-Protein Interaction (PPI) network, we submitted the identified common genes above to String online database (https://string-db.org/). Cytoscape 3.7.2 software equipped with cytoNCA plugin was used to screen out the core targets of astilbin in the treatment of RA. At the same time, the confidence level of each node and the degree of correlation between the nodes are analyzed. Further, Gene Ontology (GO) enrichment and Kyoto Encyclopedia of Genes and Genomes (KEGG) enrichment of cross-targets were conducted by R software to explorate the potential signaling pathway of astilbin in the treatment of RA.

### 2.3 Molecular docking

Firstly, the crystal structure of the core target was downloaded from the PDB Database (https://www.rcsb.org/), then the 2D structure of astilbin was obtained from the PubChem database (https://pubchem.ncbi.nlm.nih.gov) and converted to 3D structure using Chemdraw. The target protein ligand and receptor molecules were processed by Pymol software and Autodock Tools. Finally, molecular docking was accomplished using Autodock Vina and Python scripts. In addition, we calculated vina scores, with lower vina values indicating a higher affinity between the receptor and ligand. In the presence of vina less than or equal to -5, astilbin was considered to bind the target effectively.

### 2.4 Mendelian randomization analysis

To further clarify the correlation between genes and RA from a genetic perspective, we conducted a series of Mendelian randomization (MR) analyze. Summary statistics from genome-wide association studies (GWAS) for RA were obtained from the FinnGen consortium (release R12; https://www.finngen.fi/en) (19). Expression quantitative trait locus (eQTL) data were derived from the Genotype-Tissue Expression (GTEx) project (www.gtexportal.org) and the eQTLGen consortium (https://eqtlgen.org/), whereas protein quantitative trait locus (pQTL) data were sourced from the Fenland study (20, 21).

**Table 1** summarizes the GWAS datasets included in the analysis. SNPs strongly associated with eQTL were selected as instrumental variables (IVs). To ensure independence, SNPs in linkage disequilibrium were pruned (*P* > 5 × 10^-8^, r² > 0.001, window size = 10,000 kb). Weak IVs (F < 10) were excluded, and heterogeneity and horizontal pleiotropy were assessed. To minimize bias from reverse causation, Steiger filtering was applied. Colocalization analysis was performed on two-sample MR results derived from eQTL data, with cis-region defined as ±100 kb around each gene. A posterior probability of shared causality (PPH4 > 0.8) was considered evidence of a common variant influencing both gene expression and RA. Genes passing this threshold were further evaluated using summary-data-based MR (SMR) and the heterogeneity in dependent instruments (HEIDI) test, with SNPs located within ±500 kb of the gene used as IVs. All analyses were conducted in RStudio using the TwoSampleMR and MR-PRESSO packages.

**Table 1:** Data sources in this study.

Datasets	Sample	People	Consortium	Download site
The cis-eQTL in blood	31,684 670	European European	eQTLGen GTEx	https://eqtlgen.org/ https://gtexportal.org/home/datasets.
The cis-eQTL in skeletal	706	European	GTEx	https://gtexportal.org/home/datasets.
The cis-eQTL in nerve tibial	532	European	GTEx	https://gtexportal.org/home/datasets.
The cis-eQTL in cultured fibroblasts	483	European	GTEx	https://gtexportal.org/home/datasets.
The cis-eQTL in EBV transformed lymphocytes	147	European	GTEx	https://gtexportal.org/home/datasets.
Cis-pQTL in blood	4,907	European	Ferkingstad.E, et al., 2021	PMID: 34857953
RA	331,429	European	FinnGen	https://www.finngen.fi/en

### 2.4 Animals

Thirty-six female DBA mice aged 6–8 weeks with body weight of 18 ± 2 g were purchased from Vital River Laboratory Animal Technology Co., Ltd., Beijing. All animals were housed under SPF conditions at a constant temperature of 24 °C with a 12-hour light-dark cycle, and had free access to standard feed and drinking water throughout the rearing period. Prior to the formal experiment, all mice received one week of acclimatization. All animal-related procedures including model establishment, drug administration and tissue sampling were implemented in strict accordance with institutional guidelines for laboratory animal welfare and ethics. The animal experimental protocol was reviewed and approved by the Ethics Committee of Guang’anmen Hospital, China Academy of Traditional Chinese Medicine (No: IACUC-GAMH-2019-001).

### 2.5 Preparation of reagents and drugs

Bovine type II collagen (Cat. No. 20022) and complete Freund’s adjuvant (Cat. No. 7009) were obtained from Beijing Baierdi Biotechnology Co., Ltd. astilbin was supplied by Chengdu Purifa Biotechnology Co., Ltd. (Batch No. PS000649), MTX was purchased from Shanghai Sine Pharmaceutical Co., Ltd. Primary antibodies targeting MAPK1, MAPK3, HSP90AA1, PTPN11, STAT1 and SRC were sourced from Wuhan Servicebio Technology Co., Ltd. (Cat. No. GB23303). The p-MAPK1 and p-MAPK3 primary antibody were sourced from Wuhan Servicebio Technology Co., Ltd. (Cat. No. GB113492-100). The TRAP staining kit (Cat. No. G1050) was also acquired from Wuhan Servicebio, and the HE staining kit (Cat. No. G1120) was bought from Beijing Solarbio Science & Technology Co., Ltd. ALT (Cat. No. C009-2-1), AST (Cat. No. C010-2-1), BUN (Cat. No. C013-3-1) and Crea (Cat. No. C011-2-1) commercial assay kits were bought from Nanjing Jiancheng Bioengineering Institute.

Appropriate astilbin powder was weighed and dissolved thoroughly with a small volume of absolute ethanol via vortex mixing and ultrasonication. Sterile normal saline was then added dropwise under continuous stirring to prepare three working concentrations of 0.5 mg/mL, 1 mg/mL and 2 mg/mL. The prepared solutions were sterilized with a 0.22 μm aqueous filter and stored away from light. For the positive control drug, 5 mg MTX powder was dissolved in 84 mL normal saline to yield a 0.06 mg/mL stock solution, which was preserved at 4 °C in the dark.

### 2.6 CIA model establishment and treatment

On ice, bovine type II collagen and complete Freund’s adjuvant were blended thoroughly at a volume ratio of 1:1 using a three-way stopcock until complete emulsification, generating an antigen emulsion with a final collagen concentration of 2 mg/mL. Each mouse received a primary subcutaneous injection of 0.2 mL emulsion at the base of the tail and dorsal regions. A booster injection with the same volume of antigen was administered at the identical sites 21 days later. The emergence of paw swelling and erythema from day 21 post-booster immunization indicated successful establishment of the CIA model.

Thirty-six model mice were randomly allocated into six groups (n=6 per group): normal group, model group, MTX group (1.2 mg/kg/w), low-dose astilbin group (Astilbin-L, 5 mg/k/d), medium-dose astilbin group (Astilbin-M, 10 mg/kg/d), and high-dose astilbin group (Astilbin-H, 20 mg/kg/d) (12). Mice in the normal and model groups were intragastrically administered 0.2 mL normal saline once daily. Animals in the three astilbin groups received daily intragastric gavage of 0.2 mL corresponding astilbin solution, whereas the MTX group was given 0.2 mL MTX solution via intragastric administration twice weekly (22).

After four weeks of continuous treatment, all mice were anesthetized for blood collection. Whole blood samples were left undisturbed for 4 h, followed by centrifugation at 3000 rpm for 15 min. The separated serum supernatants were transferred to cryotubes and preserved at −80 °C for subsequent elisa measurement. Bilateral hind paws were dissected under clean conditions. One portion of paw tissues was fixed in paraformaldehyde for histopathological staining and micro-CT scanning; the remaining tissues were placed in cryotubes and stored at −80 °C to prepare for WB analysis.

### 2.7 Body weight, paw swelling degree and micro-CT

General conditions of mice were monitored daily, including mental status, hair appearance, food intake, water consumption, defecation and urination. Body weight was recorded every 7 days. Meanwhile, the ankle diameter of bilateral hind limbs was measured weekly with a vernier caliper, and the average value was calculated for each mouse.

Dissected hind paws were immobilized in polystyrene tubes and placed into the micro-CT scanner prior to activation of the X-ray source for survey scanning. The region of interest (ROI) within the imaging field was localized, followed by adjustment of standard pixel parameters and filters. Scanning was then conducted to acquire data for subsequent 3D reconstruction and quantitative analysis. Multiple bone microstructural indicators were compared across groups, namely bone volume (BV), bone surface (BS), BS/BV ratio, trabecular number (Tb.N), trabecular thickness (Tb.Th), and trabecular separation (Tb.Sp).

### 2.8 Pathological examination and serum ELISA assay

Mouse paw and liver tissues were immersed in universal tissue fixative for 48 h, followed by a 6-week decalcification procedure. After complete decalcification, specimens were embedded in paraffin and sectioned sequentially. Serial sections were subjected to HE staining, TRAP staining, and MAPK3 immunofluorescence staining. Tissue morphological features were observed and images were captured under an optical microscope.

Serum samples stored at −80 °C were thawed and equilibrated to room temperature. Concentrations of interleukin (IL)-1β, IL-6, IL-10, IL-17 and tumor necrosis factor-α (TNF-α) were quantified by elisa following the manufacturer’s protocols. To further evaluate the safety of astilbin, serum biochemistry was performed to assess liver and renal function indicators, including ALT, AST, Crea and BUN.

### 2.9 WB, qPCR and immunofluorescence

Synovial tissues isolated from mouse hind paws were fully lysed in RIPA buffer for total protein extraction, followed by immediate protein concentration quantification. Proteins were separated via 10% SDS-PAGE electrophoresis and electrotransferred onto PVDF membranes. After protein transfer, the membranes were blocked with 5% skim milk solution at room temperature to eliminate non-specific binding. Subsequently, the membranes were incubated with corresponding primary antibodies targeting specific proteins. Following three rounds of TBST rinsing, secondary antibody incubation was performed. β-actin was utilized as the internal control for normalizing target protein expression levels.

Total RNA was isolated from mouse synovial specimens using TRIzol reagent. Purified RNA was reverse-transcribed into complementary DNA (cDNA) in strict accordance with the operating protocols of the reverse transcription kit. The mRNA expression levels of key target genes were subsequently determined by real-time quantitative PCR with SYBR Green PCR master mix. The PCR amplification procedure was set as follows: an initial pre-denaturation step at 94 °C for 2 min, followed by 40 amplification cycles consisting of 94 °C for 10 s, 60 °C for 1 min, and 72 °C for 30 s. GAPDH was adopted as the reference gene, and the relative mRNA expression of target genes was calculated using the 2 method. The detailed primer sequences for all detected genes are listed in **Table 2**.

**Table 2:** qRCR primer sequence.

Gene	Direction	Base sequence	Primer length/bp
MAPK1	Forward Reverse	5’-ACCTCAAGCCTTCCAACCTC-3’ 5’-CTACGTACTCTGTCAAGAACCCTGT-3’	120
MAPK3	Forward Reverse	5’-AGGGCTACACCAAATCCATCG-3’ 5’-CGCTTGTTTGGGTTGAAGGTTA-3’	295
HSP90AA1	Forward Reverse	5’-CTTCTGGGGACGAGATGGTTTC-3’ 5’-CGTTCCACAAAGGCGGAGTTA-3’	124
STAT1	Forward Reverse	5’-TGCCTATGATGTCTCGTTTGC-3’ 5’-ATCTGTACGGGATCTTCTTGGA-3’	165
PTPN11	Forward Reverse	5’-GGCTCGTTAAAATGTGCCCAG-3’ 5’-GTCAGGCCATGTGGTGTAGT-3’	195
SRC	Forward Reverse	5’-AGATCACTAGACGGGAATCAGAGC-3’ 5’-GCACCTTTTGTGGTCTCACTCTC-3’	94
β-actin	Forward Reverse	5’-GTGACGTTGACATCCGTAAAGA-3’ 5’-GTAACAGTCCGCCTAGAAGCAC-3’	287

### 2.8 Statistical analysis and graph plotting

The data were presented as mean ± SD and analyzed using GraphPad Prism 10.0. ONE WAY ANOVA was used to identify different groups. *P* < 0.05 was considered to be statistically significant.

## 3 Results

### 3.1 Network pharmacology prediction and PPI analysis

**Figure 1:**
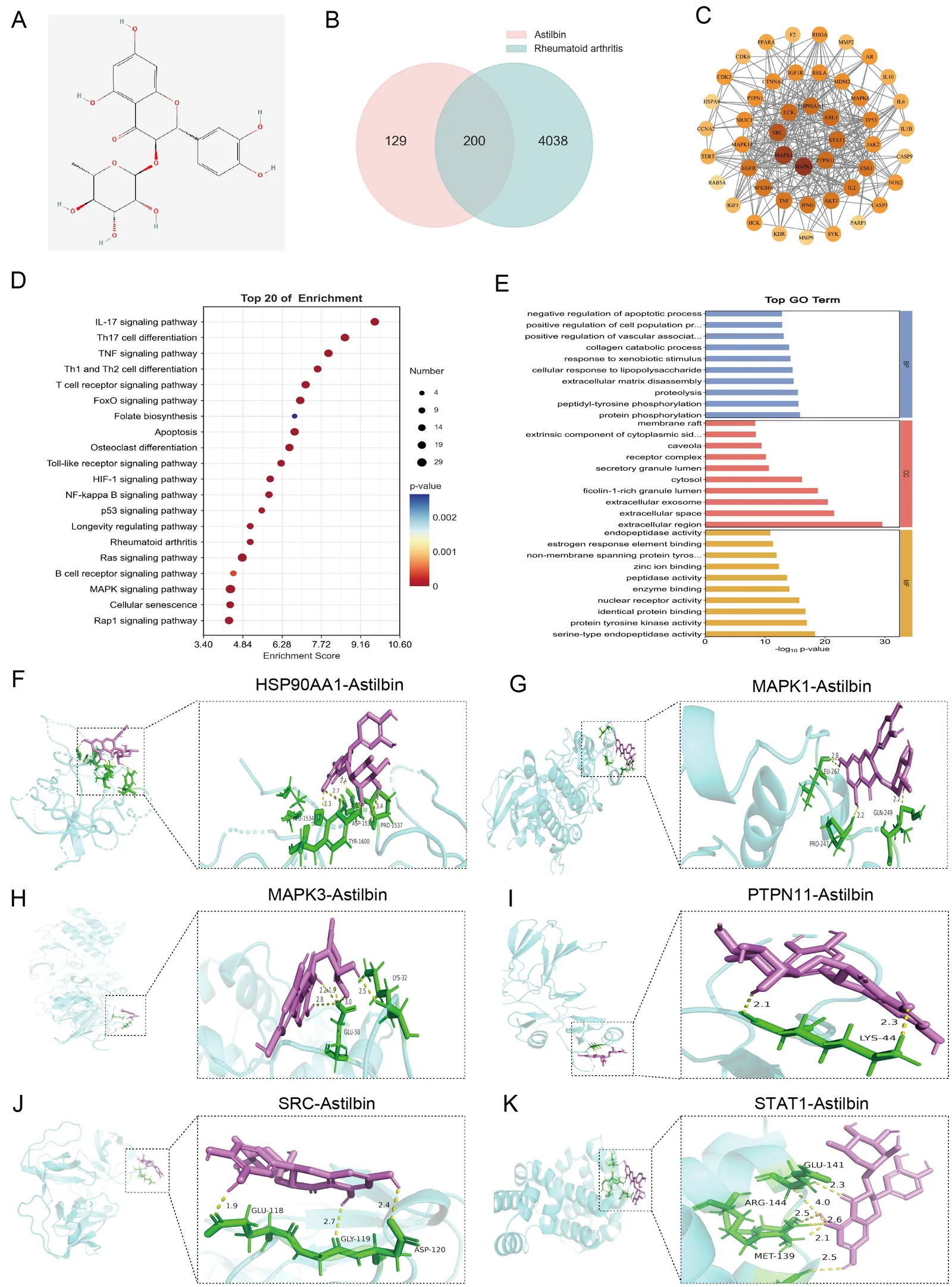
Network pharmacology and bioinformatic analyses of astilbin against RA. **(A)** Two-dimensional chemical structure of astilbin. **(B)** Venn diagram displaying the intersection of therapeutic targets of astilbin and pathogenic targets related to RA; a total of 200 overlapping targets were identified. **(C)** protein-protein interaction (PPI) network of the 48 hub targets. **(D)** Top 20 enriched pathways from KEGG enrichment analysis of overlapping targets. **(E)** GO enrichment analysis of overlapping targets, presenting the top 10 entries for biological process, molecular function and cellular component, respectively. **(F-K)** Molecular docking conformations of astilbin bound to six hub proteins, including HSP90AA1, MAPK1, MAPK3, PTPN11, SRC and STAT1.

The two-dimensional chemical structure of astilbin was retrieved from PubChem (**Figure 1A**). Four databases including the Comparative Toxicogenomics Database, PharmMapper, SwissTargetPrediction and the Traditional Chinese Medicine Systems Pharmacology Database were used to screen potential targets of astilbin. After duplicate removal, a total of 329 drug-related targets were obtained. Three databases (Therapeutic Target Database, PharmGKB and Online Mendelian Inheritance in Man) were adopted to collect RA-associated genes, yielding 4238 disease-related genes after deduplication. The overlapping targets between astilbin-related targets and RA pathogenic genes were screened and visualized via a Venn diagram (**Figure 1B**), and 200 intersecting targets were identified as candidate therapeutic targets. This result indicated that astilbin might exert therapeutic effects against RA by regulating these 200 targets.

To further explore the potential protein-protein interactions among the 200 candidate targets, PPI network analysis was performed. The gene list was imported into the STRING database to construct a PPI network consisting of 200 nodes and 719 edges, with an average node degree of 7.19. Forty-eight targets exhibited higher values of betweenness centrality (BC), closeness centrality (CC) and node degree than the overall average. The PPI network was visualized using Cytoscape software. As shown in **Figure 1C**, the top six hub targets of astilbin for RA treatment were MAPK3, MAPK1, PTPN11, HSP90AA1, STAT1 and SRC.

### 3.2 KEGG and GO enrichment analyses

To systematically elucidate the biological functions and involved signaling cascades of the 200 candidate targets through which astilbin treats RA, KEGG and GO enrichment analyses were performed in this study. KEGG pathway enrichment revealed that the intersecting targets were markedly enriched in multiple signaling axes tightly linked to RA pathogenesis, including the MAPK signaling pathway, IL-17 signaling pathway, Th17 cell differentiation, TNF signaling pathway, Toll-like receptor signaling pathway and NF-κB signaling pathway (**Figure 1D**). GO enrichment results were categorized into three major subcategories: biological process, cellular component and molecular function. In terms of biological processes, the targets were predominantly enriched in protein phosphorylation, extracellular matrix disassembly, collagen catabolic process, positive regulation of vascular smooth muscle cell proliferation, cell population proliferation and apoptotic process. For cellular components, the enriched items mainly covered extracellular region, extracellular space and extracellular exosome.

As for molecular functions, the top enriched terms contained protein tyrosine kinase activity and nuclear receptor activity (**Figure 1E**).

### 3.3 Molecular docking

Molecular docking simulation was carried out to validate the binding affinity between astilbin and the six screened hub target proteins, so as to characterize the intermolecular interactions between the small-molecule ligand and target receptors. Docking outcomes demonstrated strong binding interactions between astilbin and all six core targets. Among them, MAPK3 exhibited the lowest binding energy of −8.3 kcal/mol when complexed with astilbin (**Figures 1F–K**). Three-dimensional visualizations of binding conformations illustrated that astilbin could fully insert into the active binding pocket of each target protein. Its hydroxyl groups and benzene rings formed abundant hydrogen bonds with key amino acid residues inside the pockets, while hydrophobic interactions and van der Waals forces jointly stabilized the ligand–protein complexes. Collectively, these findings verified that astilbin is capable of stably binding to RA-associated hub proteins, which molecularly explains its multi-target regulatory mechanism against pathological progression of RA.

### 3.4 MR clarifies the correlation between genes and RA

**Figure 2:**
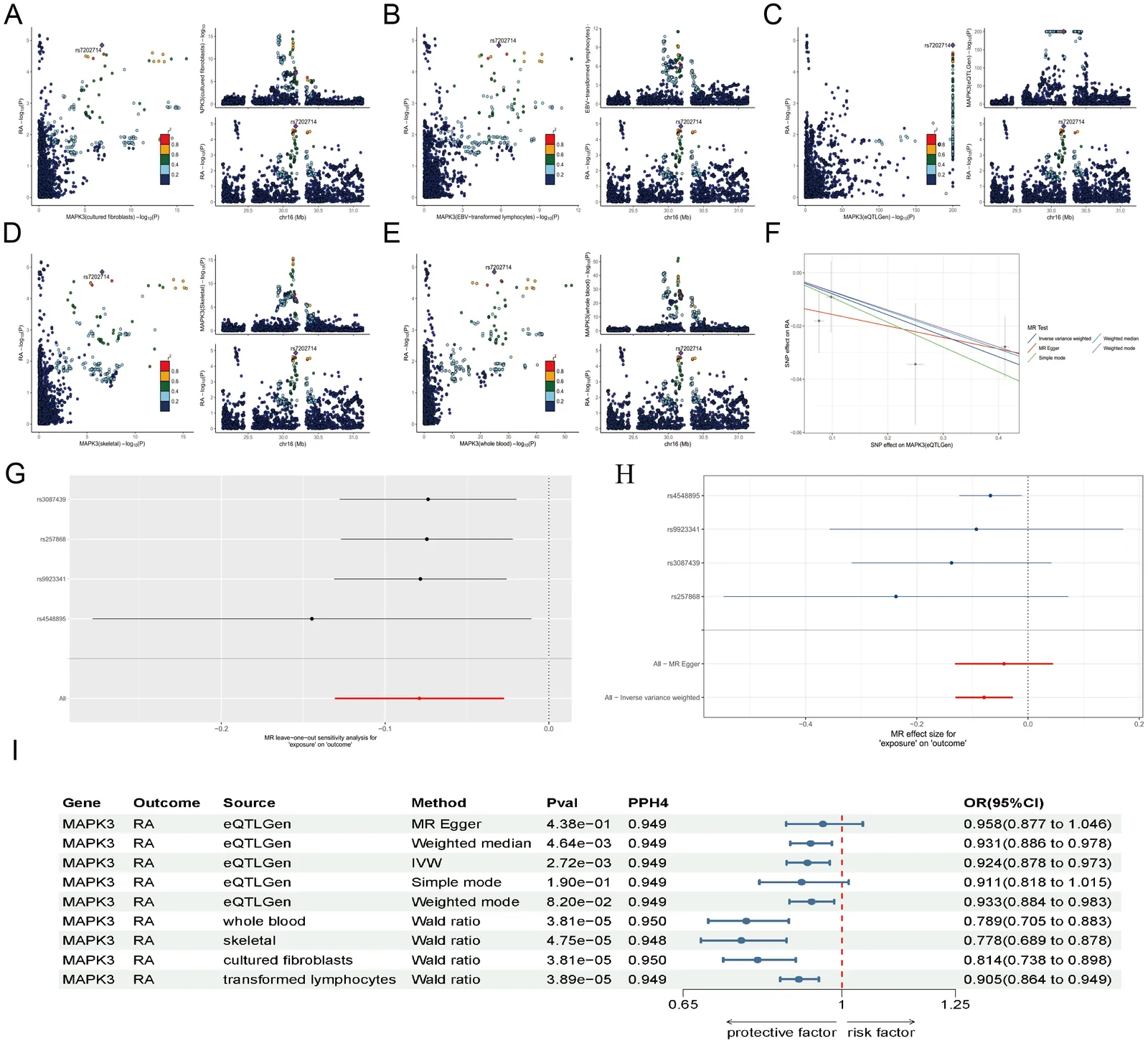
The results of Mendelian Randomization analyse. **(A-E)** Co-localization site maps for the expression of MAPK3 in cultured fibroblasts **(A)**, EBV-transformed lymphocytes **(B)**, eQTLGen **(C)**, skeletal **(D)**, and whole blood **(E)**, with RA as the outcome. **(F)** Scatter plot depicting the association between the expression of MAPK3 (eQTLGen) and RA. **(G)** Leave-one-out analysis based on eQTLGen data to evaluate the robustness of the MAPK3-RA association. **(H)** Forest plot of effect estimates for individual genetic instruments linking expression of MAPK3 (eQTLGen) to RA. **(I)** Summary forest plot integrating significant associations from two-sample Mendelian randomization and co-localization analyses across multiple tissues.

The expression of MAPK3 is significantly associated with RA across multiple tissues. In whole blood, this association was observed in both the GTEx dataset (P = 3.81 × 10^-5^; OR = 0.789; 95% CI: 0.705–0.883; PPH4 = 0.950) and the eQTLGen consortium (P = 2.72 × 10^-3^; OR = 0.924; 95% CI: 0.878–0.973; PPH4 = 0.949). Consistent associations were identified in skeletal muscle (P = 4.75 × 10^-5^; OR = 0.778; 95% CI: 0.689–0.878; PPH4 = 0.948), tibial nerve (P = 4.08 × 10^-3^; OR = 0.841; 95% CI: 0.747–0.946; PPH4 = 0.499), cultured fibroblasts (P = 3.81 × 10^-5^; OR = 0.814; 95% CI: 0.738–0.898; PPH4 = 0.950), and EBV-transformed lymphocytes (P = 3.89 × 10^-5^; OR = 0.905; 95% CI: 0.864–0.949; PPH4 = 0.949) (**Figures 2A–I**). Moreover, the direction of effect across multiple MR methods was consistent with the inverse-variance weighted (IVW) estimate, reinforcing the robustness of the observed causal relationship. At the protein level, MAPK3 remained significantly associated with RA in the SMR analysis (P < 0.05) and showed no evidence of heterogeneity in the HEIDI test (P > 0.05), suggesting that the association is likely attributable to a shared causal variant.

### 3.5 Astilbin improves general conditions and alleviates bone destruction in RA

**Figure 3:**
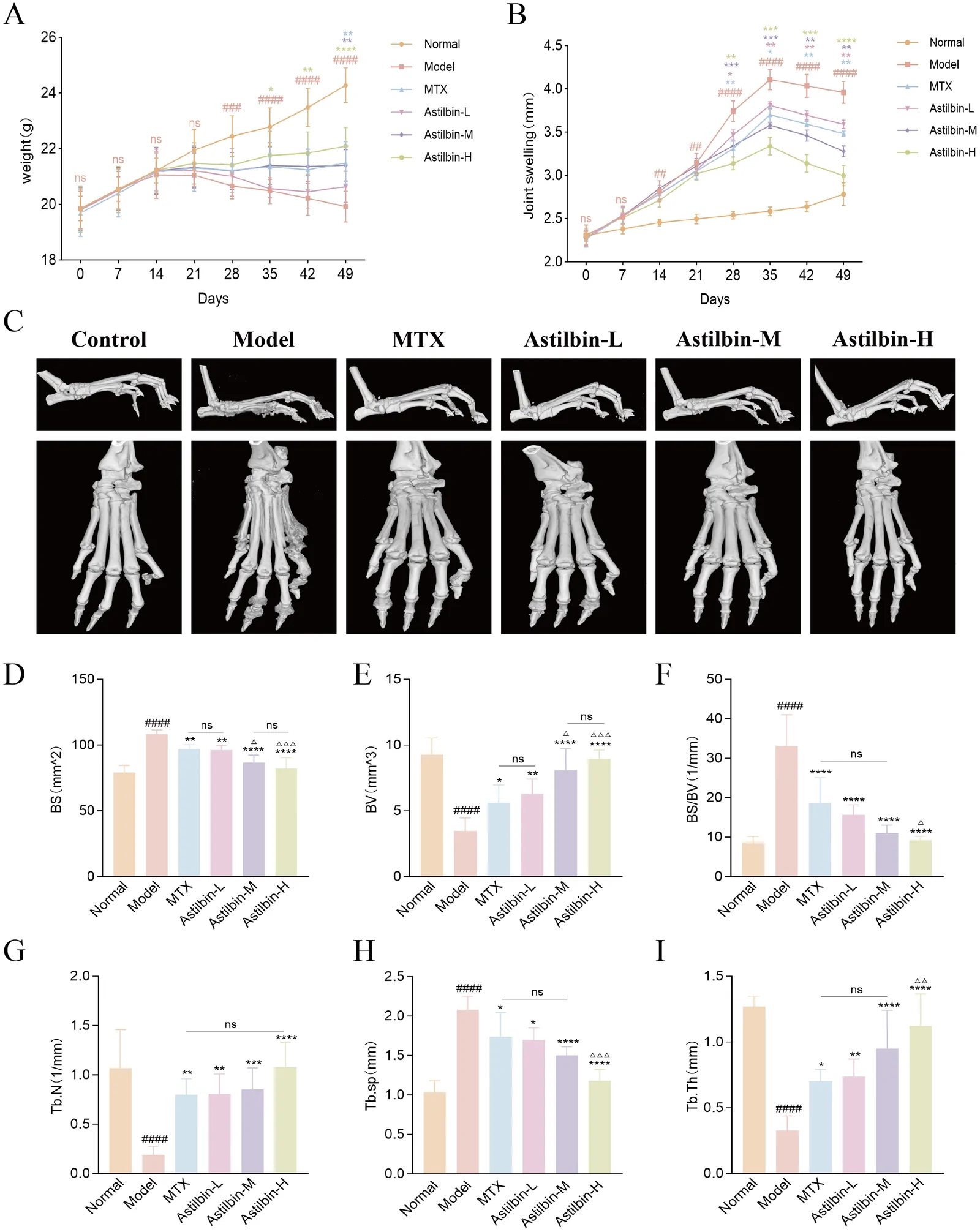
Astilbin alleviates clinical manifestations and attenuates bone destruction in CIA mice. **(A)** Dynamic body weight changes of mice at different observation time points. **(B)** Variations in hind paw thickness of mice throughout the experiment. **(C)** Representative micro-CT scanning images of mouse hind paws from each treatment group. **(D–I)** Quantitative bone microstructure parameters, including BS, BV, BS/BV, Tb.N, Tb.Sp and Tb.Th (n=6 per group). Statistical notations: ^##^*p*<0.01, ^###^*p*<0.001, ^####^*p*<0.0001, compared with Normal Group. ^*^p<0.05, ^**^p<0.01, ^***^p<0.001, ^****^p<0.0001, compared with Model Group. ^△^*p*<0.05, ^△△^*p*<0.01, ^△△△^*p*<0.001, compared with MTX Group. ns, no significance, *p*>0.05.

To validate the *in vivo* therapeutic efficacy of astilbin, CIA mouse models were established for animal functional verification. From day 7 post-modeling, model mice exhibited reduced food intake, diminished locomotor activity and retarded weight gain, with body weight markedly lower than that of normal mice. After intervention with different doses of astilbin, the mental state and mobility of CIA mice were notably recovered, and weight loss was effectively reversed. Mice receiving high-dose astilbin displayed a prominent rebound in body weight relative to the model group, indicating that astilbin ameliorates systemic wasting injury in RA models (**Figure 3A**). Continuous observation of paw swelling revealed persistent erythema, swelling and elevated skin temperature in the ankle joints of untreated model mice, whose swelling degree was significantly higher than normal group. Astilbin treatment gradually relieved ankle inflammation and swelling in a dose-dependent manner, with the most pronounced therapeutic effect observed in the high-dose group, proving that astilbin efficiently suppresses local inflammatory edema in RA (**Figure 3B**).

Micro-CT quantification of bone microarchitecture demonstrated severe bone erosion, sparse and fractured trabeculae in ankle tissues of model mice. Decreased BV, Tb.N and Tb.Th, together with elevated BS, BS/BV and Tb.Sp, collectively represented typical bone-destructive features of RA. After astilbin administration, the area of bone erosion was narrowed, trabecular bones became continuous and compact. Significant elevations in BV, Tb.N and Tb.Th as well as reductions in BS, BS/BV and Tb.Sp reflected remarkable recovery of impaired bone microstructure, confirming that astilbin mitigates RA-triggered bone resorption and structural damage (**Figures 3C–I**).

### 3.6 Astilbin ameliorates synovial hyperplasia and inflammatory responses with no apparent liver and kidney toxicity

**Figure 4:**
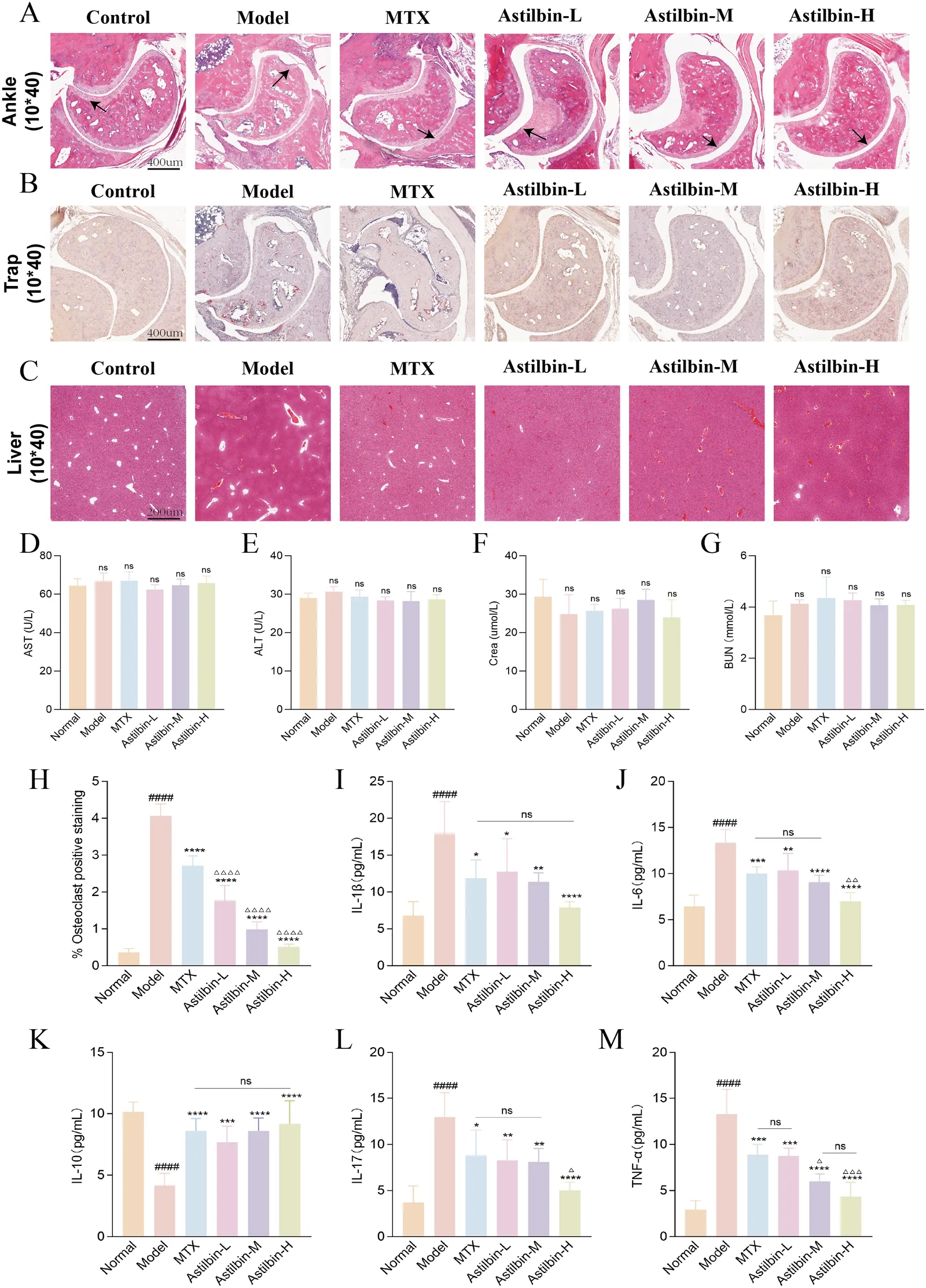
Astilbin alleviates synovial hyperplasia and inflammatory responses in RA with favorable safety profile. **(A)** HE staining of mouse hind paws. **(B)** TRAP staining of mouse hind paws. **(C)** HE staining of mouse liver tissues. **(D–G)** Serum hepatic and renal function indicators including AST, ALT, Crea and BUN in mice. **(H)** Quantification of osteoclasts in mouse hind paws (n=6). **(I–M)** Serum inflammatory cytokines IL-1β, IL-6, IL-10, IL-17 and TNF-α in mice of different groups (n=6). Statistical notations: ^####^*p*<0.0001, compared with Normal Group. ^*^p<0.05, ^**^p<0.01, ^***^p<0.001, ^****^p<0.0001, compared with Model Group. ^△^*p*<0.05, ^△△^*p*<0.01, ^△△△^*p*<0.001, ^△△△△^*p*<0.0001, compared with MTX Group. ns, no significance, *p*>0.05.

HE staining of ankle joint tissues showed intact synovial architecture in normal mice, featuring neatly arranged synoviocytes, distinct tissue layers, negligible inflammatory infiltration, regular joint space and smooth intact cartilage surface. In contrast, model mice presented obvious synovial hyperplasia and disordered cellular arrangement, accompanied by massive inflammatory cell infiltration in synovium and interstitial tissue, local congestion, edema, fibrous proliferation and irregular cartilage erosion. Astilbin treatment dose-dependently alleviated synovial hyperplasia and inflammatory cell infiltration, restoring regular tissue morphology, which demonstrated its capacity to block pathological synovial inflammation and hyperplasia in RA (**Figure 4A**). TRAP staining specifically labels activated osteoclasts, which present wine-red positive signals. Abundant TRAP-positive osteoclasts accumulated around ankle bone tissues in model mice, implying excessive osteoclast activation that drives severe bone resorption under RA pathological conditions. By contrast, astilbin intervention dramatically reduced the number and aggregation of TRAP-positive osteoclasts (**Figure 4B**). Liver sections from all astilbin-treated groups exhibited clear hepatic lobule structure and uniform hepatocyte morphology, without obvious hepatocellular degeneration, necrosis, inflammatory infiltration or fatty vacuolation, suggesting no evident hepatic toxicity of astilbin (**Figure 4C**). Furthermore, serum levels of AST, ALT, Crea and BUN in all astilbin treatment groups showed no significant differences compared with the normal group (**Figures 4D–G**).

Statistical quantification verified that all astilbin doses significantly lowered osteoclast counts and restrained abnormal osteoclast proliferation and activation compared with the model group (**Figure 4H**). Serum cytokine detection displayed drastically elevated pro-inflammatory mediators (TNF-α, IL-1β, IL-6) and suppressed anti-inflammatory IL-10 in model mice relative to normal group. Astilbin intervention markedly downregulated the secretion of TNF-α, IL-1β and IL-6 while upregulating protective IL-10, thereby restraining excessive systemic inflammatory response (**Figures 4I–M**).

### 3.7 Astilbin ameliorates RA via multi-target regulation of key signaling proteins

**Figure 5:**
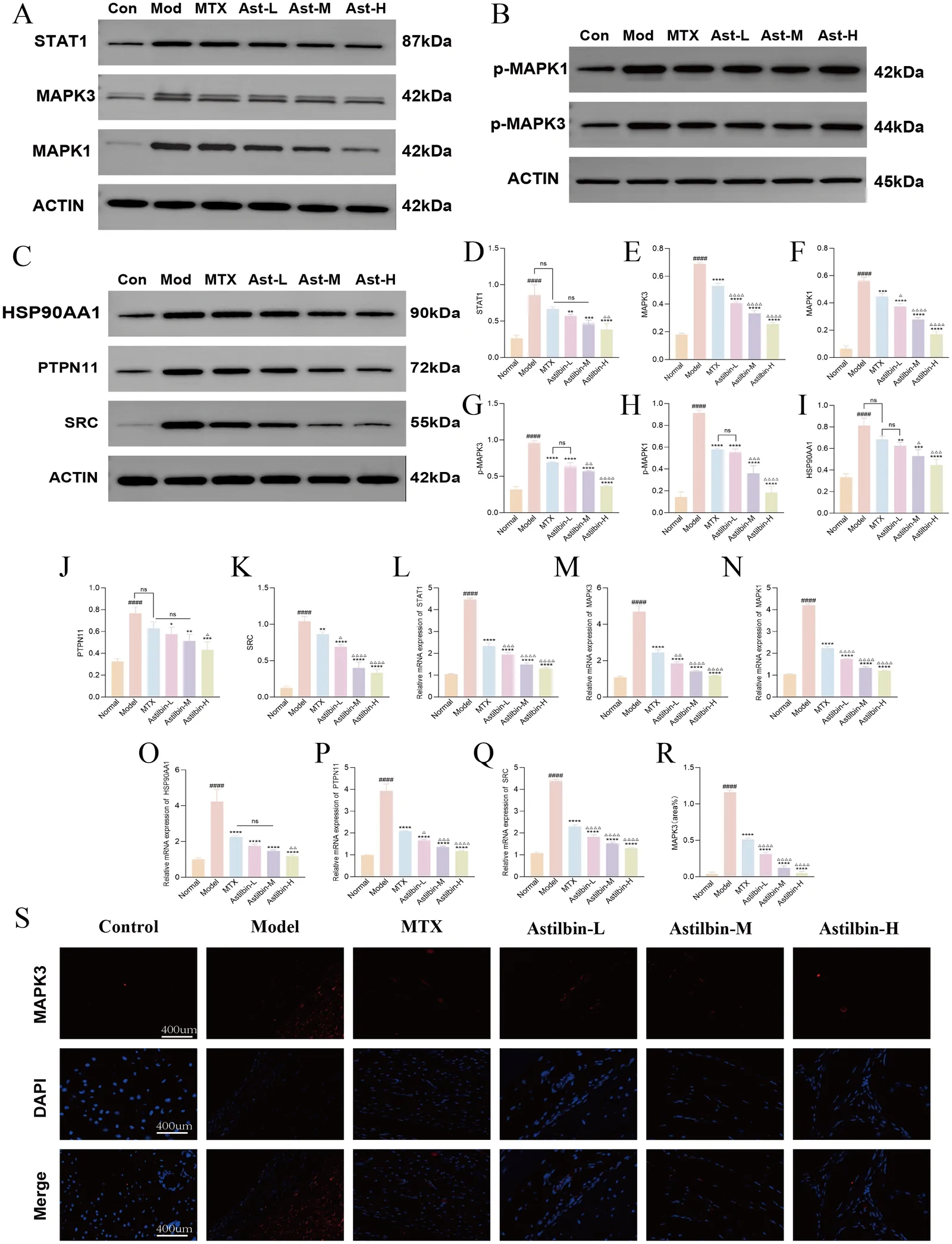
Astilbin ameliorates RA lesions via multi-target modulation of pivotal signaling proteins. **(A)** Western blot (WB) images of STAT1, MAPK3, and MAPK1. **(B)** WB images of p−MAPK3 and p−MAPK1. **(C)** WB images of HSP90AA1, PTPN11, and SRC. **(D–K)** Quantitative analysis of STAT1, MAPK3, MAPK1, p−MAPK3, p−MAPK1, HSP90AA1, PTPN11, and SRC (n=3 per group). **(L–Q)** Quantitative analysis of mRNA levels of STAT1, MAPK3, MAPK1, HSP90AA1, PTPN11, and SRC (n=3 per group). **(R)** Quantitative analysis of MAPK3 based on immunofluorescence staining. **(S)** Immunofluorescence staining for MAPK3. Statistical notations: ^####^*p*<0.0001, compared with Normal Group. ^*^p<0.05, ^**^p<0.01, ^***^p<0.001, ^****^p<0.0001, compared with Model Group. ^△^*p*<0.05, ^△△^*p*<0.01, ^△△△^*p*<0.001, ^△△△△^*p*<0.0001, compared with MTX Group. ns, no significance, *p*>0.05.

To further elucidate the potential molecular mechanism by which astilbin treats RA, WB was conducted to detect the protein levels of core targets MAPK3, MAPK1, PTPN11, HSP90AA1, STAT1, SRC, as well as the phosphorylation levels of MAPK1 and MAPK3 (**Figures 5A–C**). WB results demonstrated that the protein expression of MAPK3, MAPK1, PTPN11, HSP90AA1, STAT1 and SRC was significantly increased in joint tissues of the model group compared with the normal control group. Different doses of astilbin markedly downregulated the aberrant expression of the above core proteins and inhibited excessive activation of these key proteins, exerting prominent regulatory effects on the corresponding targets (**Figures 5D–K**).

Furthermore, PCR was performed to verify the mRNA expression levels of MAPK3, MAPK1, PTPN11, HSP90AA1, STAT1 and SRC, and the PCR results were consistent with the WB data. The mRNA levels of these six genes were significantly upregulated in joint tissues of CIA model mice. After astilbin intervention, the abnormally high expression of these core genes was significantly decreased in a dose-dependent manner, suggesting that astilbin inhibits aberrantly activated core target genes in RA at the transcriptional level (**Figures 5L–Q**).

Immunofluorescence staining was performed to visually verify the expression shift of the representative hub protein MAPK3. The fluorescence intensity and positive staining area of MAPK3 were greatly increased in joint tissues of model mice, reflecting massive activation of MAPK3 under RA pathological conditions. In contrast, astilbin treatment remarkably weakened MAPK3 fluorescence signals and shrank its positive staining region, further validating that astilbin effectively inhibits the excessive expression of MAPK3 (**Figures 5R, S**).

## 4 Discussion

RA is a chronic progressive autoimmune disease driven jointly by multiple factors including genetic susceptibility, environmental stimuli and disrupted immune homeostasis (23, 24). The disease follows a prolonged and recurrent clinical course, with persistent inflammatory infiltration of joint synovium as a core early hallmark (25). As the major bioactive flavonoid constituent from Smilax glabra Roxb., astilbin has been validated to exert extensive pharmacological activities such as anti-inflammation, anti-oxidation and modulation of cell proliferation and differentiation (26, 27). Nevertheless, the regulatory network and core functional mechanisms of astilbin in RA-associated bone destruction have not been systematically elucidated. Considering the existing gaps in current research, this study integrates network pharmacology target screening, molecular docking simulation and experiments in CIA mice. Systematic validation was conducted focusing on the therapeutic targets, signaling pathways, *in vivo* pharmacodynamic efficacy and safety of astilbin against bone destruction in RA. This work aims to provide novel molecular mechanisms and candidate natural bioactive agents for targeted intervention of RA-induced bone injury.

Bone destruction represents an irreversible landmark pathological outcome during RA progression, leading to permanent joint deformity, loss of limb motor function and severely impaired long-term quality of life in patients (28, 29). In this study, Micro-CT three-dimensional reconstruction quantitative technology was adopted to achieve precise quantitative evaluation of trabecular microarchitecture in mouse ankle joints. The results revealed typical erosive bone lesions in untreated CIA mice. Protective bone microstructural indices including BV, Tb.N and Tb.Th were markedly decreased, whereas BS/BV and Tb.Sp were significantly elevated. The trabeculae became sparse, thin, fractured and defective with extensive bone resorption cavities. In contrast, continuous astilbin treatment preserved better trabecular continuity, reduced the area of bone cavities, and improved all quantitative bone microstructural parameters in a dose-dependent manner. TRAP-specific staining demonstrated massive aggregation and fusion of mature wine-red osteoclasts along the cortical bone and trabecular edges in model mice, indicating drastically enhanced osteoclast proliferation and differentiation. After astilbin administration, the number of TRAP-positive osteoclasts was significantly reduced, and fused osteoclast clusters were obviously alleviated. These findings confirm that astilbin suppresses osteoclast proliferation, fusion and terminal differentiation, blocks bone resorption, and thereby exerts osteoprotective effects.

Uncontrolled persistent synovitis acts as the upstream core driver initiating bone destruction in RA (30). In the early stage of the disease, immune tolerance is disrupted. Abnormally activated peripheral T cells and macrophages extensively infiltrate the joint synovium, driving excessive differentiation of Th17 cells. Robust secretion of pro-inflammatory cytokines such as TNF-α, IL-6 and IL-17 establishes a sustained local pro-inflammatory microenvironment (31, 32). Long-term inflammatory signaling continuously acts on stromal cells and osteoclast precursors surrounding bone tissue, persistently triggering osteoclast differentiation programs (33, 34). Massive mature osteoclasts are generated to degrade bone matrix, ultimately resulting in irreversible bone defects. Serum ELISA results in the present study directly verified the potent anti-inflammatory capacity of astilbin. Compared with normal control mice, CIA model mice exhibited dramatically elevated serum levels of pro-inflammatory cytokines TNF-α, IL-1β and IL-17, accompanied by markedly reduced secretion of the protective anti-inflammatory cytokine IL-10, indicating thorough disruption of systemic inflammatory balance. Astilbin administration dose-dependently lowered the levels of various pro-inflammatory mediators and simultaneously restored IL-10 expression, effectively reconstructing inflammatory homeostasis *in vivo*. Meanwhile, HE staining of ankle joint pathological sections showed that astilbin treatment markedly relieved synovial hyperplasia, reduced the number of infiltrated inflammatory cells, and restored regular arrangement of synovial cells. Collectively, these data demonstrate that astilbin inhibits excessive inflammatory proliferation in both synovium and systemic circulation, interrupts sustained release of inflammatory signals, and fundamentally attenuates inflammation-driven bone destruction.

As a core intersecting pathway linking inflammation and bone metabolism, excessive activation of the MAPK signaling pathway serves as a key molecular trigger for sustained synovial inflammation and progressive bone destruction in RA (35, 36). In early RA, abundant pro-inflammatory cytokines sequentially activate Raf-MEK kinases, ultimately inducing threonine and tyrosine phosphorylation of MAPK1 and MAPK3, which converts them from inactive resting forms into functionally activated proteins (37, 38). On one hand, activated MAPK complexes continuously amplify local synovial inflammatory signaling and facilitate further production of inflammatory cytokines. On the other hand, activated MAPK translocates into the nucleus to directly initiate osteoclast-specific transcriptional programs, leading to irreversible bone destruction (39–42). Inhibition of the MAPK pathway can significantly alleviate RA pathological manifestations. In the current study, network pharmacology combined with KEGG enrichment analysis identified the MAPK pathway as a core target underlying astilbin function. Further Mendelian randomization analysis validated the tight association between MAPK3 and RA. *In vivo* detection of total MAPK1, MAPK3 protein as well as phosphorylated p-MAPK1 and p-MAPK3 substantiated the hypothesis that astilbin may treat RA via modulating the MAPK signaling pathway.

SRC, STAT1, HSP90AA1 and PTPN11, other core hub proteins screened by network pharmacology, may form a synergistic regulatory network together with MAPK3 and MAPK1. As a non-receptor tyrosine kinase, SRC amplifies upstream inflammatory signals mediated by TNF and IL-17 and further promotes MAPK3 phosphorylation (43). STAT1 mediates interferon-associated inflammatory cascades and aggravates synovial immune disturbance (44). Acting as a molecular chaperone, HSP90AA1 maintains the three-dimensional stability of MAPK proteins, prevents kinase degradation and prolongs pathway activation duration (45). PTPN11 participates in negative feedback balance regulation of this pathway (46). Astilbin can simultaneously target the above multiple molecules, comprehensively attenuating MAPK activation mediated by diverse pathways, and exert integrated pharmacological effects via multi-target synergistic suppression of inflammation and osteoclast differentiation. Notably, the present research mainly focuses on the mechanism by which astilbin regulates the MAPK signaling pathway. The synergistic effects among these targets will be explored in our follow-up investigations.

Although this study confirms that astilbin alleviates synovial and systemic inflammation as well as delays bone destruction in RA through modulation of the MAPK signaling pathway, several limitations remain. Firstly, this research only performed detections at the animal tissue level. *In vitro* functional experiments using osteoclasts and fibroblast-like synoviocytes, rescue assays with MAPK3 overexpression/knockdown, and an animal control group treated with MAPK pathway inhibitors were not implemented. Existing *in vivo* evidence can only demonstrate correlation, rather than directly proving that MAPK3 is an indispensable core target responsible for the osteoprotective effects of astilbin. Secondly, further evidence is required to support translational application from basic research to clinical practice. All Mendelian randomization analyses were based on genetic cohorts of European populations. Significant disparities exist in genetic polymorphisms among European cohorts, mice and Asian RA patients, which limits the extrapolation of genetic association conclusions. Furthermore, data regarding bioavailability and pharmacokinetics remain to be characterized. To address these limitations, subsequent experiments will be carried out using *in vitro* cell models, and MAPK pathway inhibitor groups will be added for verification. In view of the drawbacks of astilbin including poor water solubility, *in vivo* isomerization and low oral bioavailability, novel delivery strategies such as nanocarriers will be developed to improve its metabolic stability and bioavailability.

## 5 Conclusion

Astilbin may exert multiple pharmacological effects including anti-inflammation, inhibition of osteoclast activation, improvement of bone microarchitecture and alleviation of bone destruction in RA via regulating the MAPK signaling pathway, and it exhibits favorable safety *in vivo*.

## Data Availability

The original contributions presented in the study are included in the article/**Supplementary Material**. Further inquiries can be directed to the corresponding author.
